# Influence of nerve cuff channel count and implantation site on the separability of afferent ENG

**DOI:** 10.1088/1741-2552/aabca0

**Published:** 2018-05-09

**Authors:** Carolina Silveira, Emma Brunton, Sally Spendiff, Kianoush Nazarpour

**Affiliations:** 1 Intelligent Sensing Laboratory, School of Engineering, Newcastle University, NE1 7RU, United Kingdom; 2 Institute of Genetic Medicine, Newcastle University, Newcastle-upon-Tyne, United Kingdom; 3 Institute of Neuroscience, Newcastle University, Newcastle-upon-Tyne, United Kingdom; A.C.Silveira@newcastle.ac.uk; Emma.Brunton@newcastle.ac.uk; Kianoush.Nazarpour@newcastle.ac.uk

**Keywords:** sensory feedback, electroneurography, nerve cuff, classification

## Abstract

*Objective*. Recording of neural signals from intact peripheral nerves in patients with spinal cord injury or stroke survivors offers the possibility for the development of closed-loop sensorimotor prostheses. Nerve cuffs have been found to provide stable recordings from peripheral nerves for prolonged periods of time. However, questions remain over the design and positioning of nerve cuffs such that the separability of neural data recorded from the peripheral nerves is improved. *Approach*. Afferent electroneurographic (ENG) signals were recorded with nerve cuffs placed on the sciatic nerve of rats in response to various mechanical stimuli to the hindpaw. The mean absolute value of the signal was extracted and input to a classifier. The performance of the classifier was evaluated under two conditions: (1) when information from either a 3- or 16-channel cuff was used; (2) when information was available from a cuff placed either distally or proximally along the nerve. *Main results*. We show that both 3- and 16-channel cuffs were able to separate afferent ENG signals with an accuracy greater than chance. The highest classification scores were achieved when the classifier was fed with information obtained from a 16-channel cuff placed distally. While the 16-channel cuff always outperformed the 3-channel cuff, the difference in performance was increased when the 16-channel cuff was placed distally rather than proximally on the nerve. *Significance*. The results indicate that increasing the complexity of a nerve cuff may only be advantageous if the nerve cuff is to be implanted distally, where the nerve has begun to divide into individual fascicles.

## 1. Introduction

The development of prosthetic devices capable of interfacing with the human nervous system has been a popular topic of research in the past decades [1–9]. Patients suffering from spinal cord injury, limb loss and stroke could benefit from neuroprostheses able to provide motor control and sensory feedback [1–3, 5, 6, 8, 10–12]. Even though existing prosthetic solutions [6, 8, 9, 13, 14] are able to restore some function to the users, these devices cannot rival the functionality of natural limbs. As sensory feedback plays a crucial role in restoring users’ movement capability [9], neuroprostheses would significantly improve if they could provide close-to-natural sensation [3]. An important aspect of developing closed-loop neurostimulation systems is being able to identify different sensations that would normally be conveyed by the peripheral nerves to the central nervous system. One way of obtaining sensory information is to record from intact peripheral nerves.

A number of neural interfaces have been investigated for use within closed-loop prostheses [1, 5–8, 15, 16]. These interfaces have been used to record from and stimulate whole peripheral nerves aiming to create neuroprostheses that deliver sensory feedback to the user. When choosing a neural interface for a specific purpose, a number of attributes need to be considered including: invasiveness, ease of implantation, the signal to noise ratio and selectivity [2, 5–7, 15, 17, 18]. Some of these interfaces include intraneural electrodes such as the longitudinally implanted intrafascicular electrodes (LIFEs) [4, 6], the transverse intrafascicular multichannel electrodes (TIMEs) [15] and the high-density Utah slanted electrode array (HD-USEA) [18, 19]. These interfaces allow the recording and/or stimulation of nerve fibres, making them highly selective neural interfaces. On the other hand, extraneural electrodes such as nerve cuffs [20], epineural [21], book electrodes [22] and flat-interface electrodes [23] are less selective but are also less invasive [2, 7, 23]. Nerve cuffs, in particular, offer an easier implantation process than their intraneural counterparts and are shown to remain physically and functionally stable over time [2, 9, 24–29].

Numerous studies investigated the recording properties of nerve cuff electrodes. For example, Sahin and Durand [30], and Struijk *et al* [31] both showed that it was possible to identify the nerve branch where electrical stimulation was applied in nerve cuff recordings made more proximally on the nerve trunk. More recently, Raspopovic *et al* [5] and Brunton *et al* [11] demonstrated that it is possible to identify more naturally occurring neural signals in whole nerve recordings using nerve cuffs. Nevertheless, despite the wealth of the literature over the last three decades, two key questions are still not addressed:

(i) Does increasing the number of channels in a cuff aid in the separation of neural signals enough to warrant the increased complexity and cost of a cuff design?
(ii) Would the location where the cuff is placed along the nerve affect the separability of neural signals?

In this study, we sought to answer these two questions. Firstly, we implanted two neural cuffs, one with 16 contacts and the other with three contacts, on the sciatic nerve of rats to record neural signals in response to mechanical stimulation of the hindpaw. Features were extracted from the recorded neural signals and introduced to a linear classifier. The performance of the classifier with the information obtained from both cuffs was compared. Secondly, we implanted two 16-channel nerve cuffs at two different locations along the sciatic nerve, one distal and one proximal. The electroneurographic (ENG) signals in response to mechanical stimulation of the hindpaw were recorded and classified.

## 2. Methods

### 2.1. Animal preparation and surgery

In total six Sprague Dawley rats (weight: 400–480 g) were used in this study. All procedures involving animal work were performed under respective UK Home office licences following the Animals (Scientific Procedures) Act (1986) and were approved by the Animal Welfare and Ethical Review Board of Newcastle University. The animals were housed in a 12 h light/dark cycle, with food and water available *ad libitum*.

Anaesthesia was induced with an intraperitoneal (IP) injection of a combination of Hypnorm and Midazolam at 0.27 ml per 100 g [32]. Anaesthetic depth was maintained with Isoflurane in Oxygen delivered through a nose cone. Further IP injections of the Hypnorm/Midazolam cocktail were given as needed. The Isoflurane levels did not exceed 0.5% during the recording of ENG signals. At the cessation of each experiment, the animal was humanely killed with an overdose of Pentobarbitol without waking up from anaesthesia.

**Figure 1.**
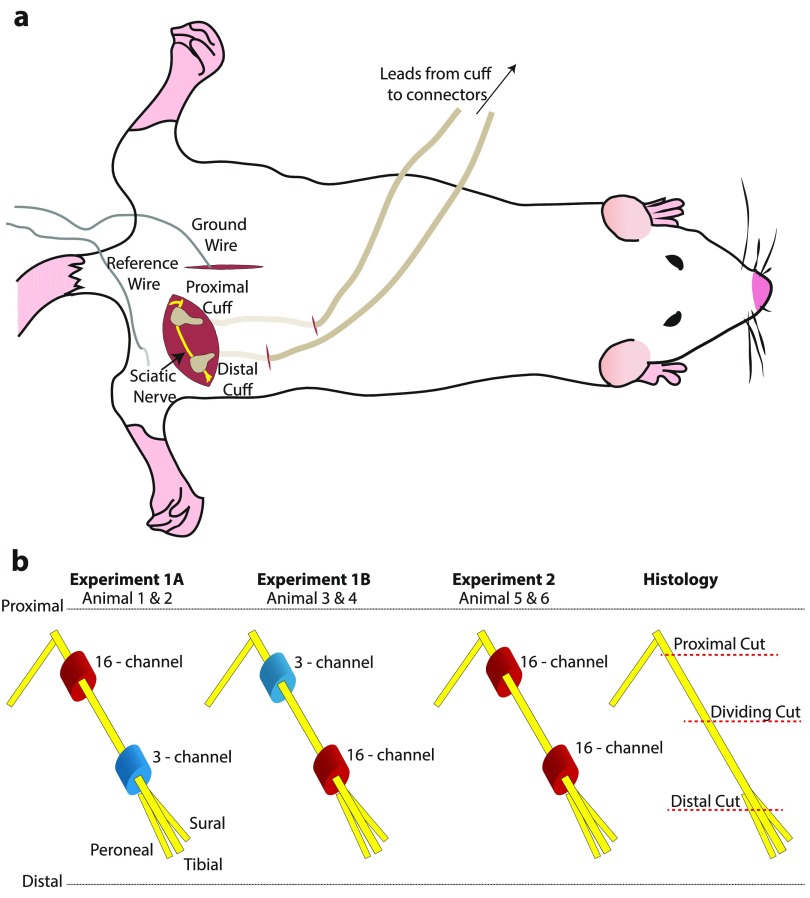
(a) Schematic representation of cuff electrodes implanted on the sciatic nerve, the ground wire placed on the L5 spinous process and the reference wire placed in the skin. The cuffs’ leads were tunnelled under the skin and muscle. (b) Position of the two cuffs on the sciatic nerve (yellow) for each of the three experiments performed. The distal position is closer to the site where the sciatic nerve divides into its peroneal, tibial and sural branches. Also shown is where the nerve was cut for histology.

An incision was made in the skin approximately 1 cm caudal and parallel to the femur. The muscle was then carefully dissected to expose the sciatic nerve. A second and a third incision were made, about 3 cm rostral to the first incision to create two parallel tunnels under the skin and the muscles. The nerve cuffs were tunnelled through the incisions and implanted on the sciatic nerve. A piece of cotton was placed in between the two cuffs to separate them by approximately 1.5 cm. Silicone sealant (Kwik-Cast, World Precision Instruments, FL, USA) was applied around the cuffs to secure them in place. A tungsten wire was wrapped around the L5 spinous process and fixed with dental cement to act as a ground. A stranded stainless steel wire placed in the skin was used as a reference (figure 1(a)).

Two different types of nerve cuffs were used in this study, namely, a 16-channel cuff and a 3-channel cuff (Microprobes for Life Science: Gaithersburg, MD, USA). The 16-channel cuff was a concentric nerve cuff of 4.25 mm in length and an inside diameter of 1.0 mm. The cuff consisted of four rings and each ring contained four electrode contacts. The 3-channel cuff was a standard nerve cuff measuring 4 mm in length and with an inside diameter of 1.0 mm with three ring contacts. In both cuffs the contacts were made from 100 *μ*m diameter platinum wire.

On the six animals, two different experiments were performed, namely Experiment 1 and Experiment 2. The two experiments were conducted independently of each other. Different protocols were applied for each experiment and each experiment aimed to answer a different question. Common to both experiments, neural signals were recorded with two cuffs placed on the sciatic nerve, one distally and the other proximally as shown in figure 1(a). The distal cuff was placed close to the site where the sciatic nerve divides into its peroneal, tibial and sural branches. The proximal cuff was placed approximately 1.5 cm proximal to the distal cuff. The arrangement of the cuffs in both experiments is described below.

#### 2.1.1. Experiment 1.

We set out to compare the separability of the ENG signals recorded simultaneously with the 3- and 16-channel cuffs. Experiment 1 comprised parts A and B, depending on the positioning of the 3- and the 16-channel cuffs on the nerve. In part A, the 3- and 16-channel cuffs were implanted distally and proximally, respectively. This arrangement was reversed in part B where the 3-channel cuff was implanted proximally and the 16-channel cuff distally in the nerve. The positioning of the cuffs is depicted in figure 1(b). Animals 1 and 2 were used in part A and Animals 3 and 4 in part B.

#### 2.1.2. Experiment 2.

In this experiment, we investigated whether the relative position of a cuff on the sciatic nerve would impact the separability of the neural data. Two 16-channel cuffs were implanted on the sciatic nerve; one proximally and one distally, as shown in figure 1(b). Animals 5 and 6 were used in this experiment.

### 2.2. Mechanical stimulation

In Experiments 1A and 1B, three types of mechanical stimulation were applied to the rat’s hindpaw: proprioception, nociception and touch. For the proprioceptive trials the rat’s hindpaw was moved, from a resting position, to six angles:  ±10°, ±20° and  ±30°. For the touch stimuli, two Von Frey fibres (100 g and 300 g in Experiment 1A, and 8 g and 15 g in Experiment 1B) were used to touch the heel of the rat. Comparisons were only made within animals and not between. Therefore, changing the type of the Von Frey fibres in different animals would not affect the results. Finally, for the nociception stimuli, the rat’s toes and heels were pinched using a pair of forceps instrumented with a force sensitive resistor (FSR). For each of the stimuli types, stimulation was applied during three seconds (stimulus ON) and then the hindpaw was kept in the rest state for another three seconds (stimulus OFF). In Experiment 1A, the nociception stimuli was applied for one second only (stimulus ON) and the interval between stimuli was also one second (stimulus OFF). Whilst we adopted the protocol described in [11] and used the same hardware for mechanical stimulation, two important refinements were made, namely:

(i) the rat’s hindpaw was attached to an aluminium rod extending from the servo motor throughout the whole procedure;
(ii) the type and order of the mechanical stimuli were pseudo-randomised.

The first refinement ensured that neural recordings would be of a similar level during the rest periods, allowing for reproducible stimuli across all trials. The latter refinement reduced the likelihood that temporal changes, such as anaesthetic depth, would influence the results. Specifically, a proprioceptive block of trials consisted of the hindpaw being moved 60 times, ten times to each angle in a pseudo-random order. For nociception and touch a block consisted of ten applications of the same stimulus. For each experiment a list containing the order and the type of stimuli was pseudo-randomly generated.

In Experiment 2 only proprioceptive stimuli were applied as this type of stimulus showed the highest classification rates in Experiment 1. In addition, this selection shortened the duration of the experiment. Experiments 1 and 2 address different questions. Since we do not directly compare between animals, changing the experimental procedure for Experiment 2 does not affect the overall conclusions.

### 2.3. Neural data recording and classification

**Figure 2.**
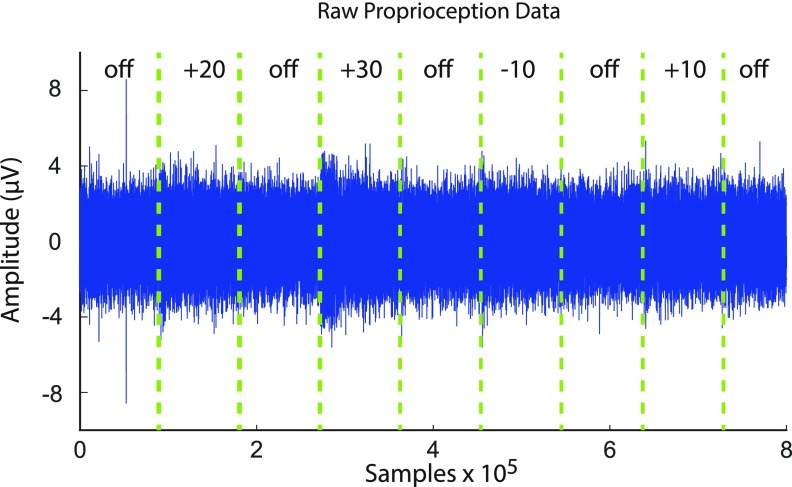
Filtered nerve signal recorded from one electrode on the 16-channel cuff during a proprioception block. The green dashed lines indicate the begin and end of each trial. The class of the signal is indicated above the nerve signal.

A Cerebus Neural Signal Processor and a Cereplex M32 head stage (Blackrock Microsystems, USA) were used to record the ENG signals at a sampling frequency of 30 kHz. The signals were then digitally filtered in MATLAB^TM^ using a finite-impulse response bandpass filter between 800 and 2200 Hz [5, 11]. In this work, we were only interested in the steady-state response, that is, when the motors were not moving. Adopting this approach, we avoided any motor movement-related artefacts in the recorded signals. Thus, after the data was filtered, the times when stimuli were applied were identified using comments automatically recorded in the proprioception and touch files. Figure 2 illustrates the filtered nerve signals. The dashed lines indicate the start and end of the proprioceptive classes. For the nociception stimuli the signal recorded from the FSR sensor was used to identify the stimulus ON and OFF times. A window of 0.5 s was used for feature extraction beginning 0.25 s after stimulus onset was identified. This window was extracted for all the stimuli of Experiments 1A and 1B. This window was chosen since there was only one second of nociception stimulus application in Experiment 1A. For Experiment 2, a window of 2.5 s was used beginning 0.25 s after stimulus onset identification.

To measure the separability of the data, we extracted the mean absolute value (MAV) of the ENG signal from each channel during the stimulus ON times. The MAV feature has been extensively used in research and clinical practice to extract information from ENG signals [5, 11] because it is computationally inexpensive and it provides better results than other features [5]. We input this feature vector into a linear discriminant analysis (LDA) classifier. Comparative feature and classifier analysis falls outside the remit of this paper.

In Experiment 1, there were ten mechanical stimulation classes, namely, six proprioception angles (±10°, ±20° and  ±30°), two nociception sites (heel and toe pinch) and two touch classes (8 g and 15 g Von Frey fibres). We collected 50 samples for each stimulus. In Experiment 2, only the six proprioceptive stimuli were applied. Therefore, a total of 300 samples, per animal, were collected. A very small number of samples were removed from analysis, due to measurement noise. In all cases we report balanced classification scores, i.e. we corrected for any imbalance in the number of trials within each class [33].

### 2.4. Cross-validation and visualisation

A five-fold cross-validation was used to explore how the classifier performed on unseen data. The validation accuracy obtained corresponded to the median of the percentages of correctly classified instances of the five folds. The difference between the performance of the two cuffs was also calculated for each fold. To visualise the feature space, all possible combinations of electrode pairs were compared in terms of the cross-validation accuracies achieved with an LDA classifier. The normalised MAVs of the best performing pair of electrodes were scattered plotted. Finally, the cross-validated confusion matrices were generated for better visualisation of the obtained results.

### 2.5. Histology

The sciatic nerve was removed from the animal and cut into two parts, namely, distal and proximal, as illustrated in the rightmost section of figure 1(b). Both the distal and proximal parts of the nerve were frozen in liquid nitrogen pre-cooled isopentane and mounted on cork discs. Then, 10 *μ*m cryosections were cut using a CM1860 cryostat (Leica) at  −25 °C, mounted on Histobond adhesion microscope slides, and stored at  −80 °C until use. Sections for histology were air dried for 1 h before being immersed in haematoxylin for 1 min. Sections were then washed in running tap water for 2 min and then immersed in eosin for 30 s before a final wash in tap water. Sections were taken through a dehydrating graded ethanol series (75%, 95%, 100%  ×  2), and cleared in two rounds of histoclear before mounting using DPX. Tiled images were taken using a Zeiss Axio Imager fluorescent microscope with Zen software at  ×20 magnification.

## 3. Results

### 3.1. Comparison of 3- and 16-channel cuffs

**Figure 3.**
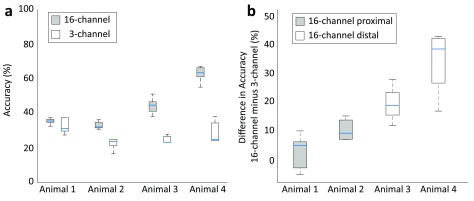
(a) Five-fold cross-validated classification accuracy obtained in Experiments 1A and 1B for Animals 1 to 4. Chance level: 10% (ten stimulation classes). (b) Difference in classification score in each fold for Animals 1–4. Box plots show median scores across ten classes within each animal. Straight lines, medians; solid boxes, interquartile ranges; whiskers, overall ranges of non-outlier data.

The ability of the classifier to discriminate different neural signals using features extracted from either the 3- or the 16-channel nerve cuff was compared. Figure 3(a) presents the cross-validated classification accuracies. In both parts of Experiment 1 and in all four animals, the median accuracy using the ENG signals from the 16-channel cuff was higher than that achieved when using the ENG signals from the 3-channel cuff. However, the difference between the classification scores for the 3- and 16-channel cuffs was much greater when the 16-channel cuff was placed distally (figure 3(b)).

The accuracies obtained with the 3- and 16-channel cuffs in Experiment 1A were comparable. The median difference between the performance of the classifier when information was used from the 16-channel versus the 3-channel cuff was 5.0% and 9.3% for Animal 1 and 2, respectively (figure 3). In some folds in Animal 1, the 3-channel cuff even outperformed the 16-channel cuff (figure 3(b)). By comparison, in Experiment 1B where the position of the cuffs along the nerve was swapped, the improvement in performance of the classifier when fed with information from the 16-channel cuff compared to the 3-channel cuff was increased. The median difference between the performance of the classifier when using information from the 16- compared to the 3-channel cuff was 19.0% and 38.6% for Animals 3 and 4, respectively.

**Figure 4.**
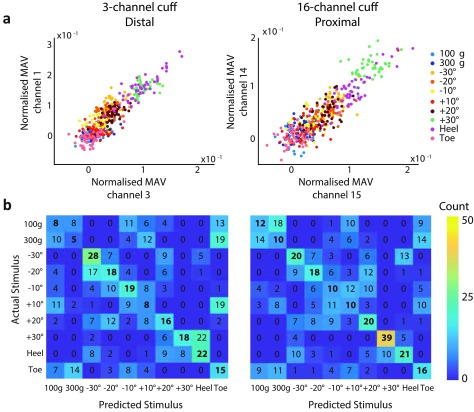
Experiment 1A—example results from Animal 1; (a) scatter plots presenting the best-performing pair of features for the 3- (left) and 16-channel (right) cuffs; (b) confusion matrices for classification of the ENG data recorded with the 3- (left) and 16-channel (right) cuff electrodes.

**Figure 5.**
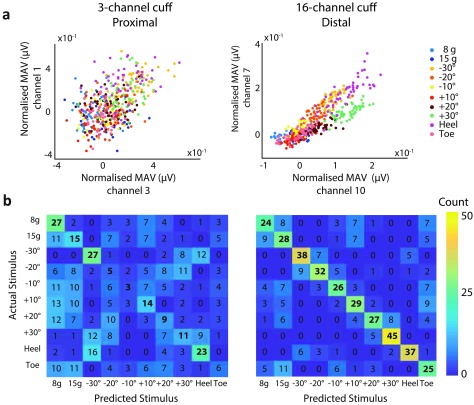
Experiment 1B—example results from Animal 4; (a) scatter plots presenting the best-performing pair of features for the 3- (left) and 16-channel (right) cuffs; (b) confusion matrices for classification of the ENG data recorded with the 3- (left) and 16-channel (right) cuff electrodes.

To help elucidate the difference in the performance of the classifier when fed with information from the different cuffs, we examined scatter plots of the feature space and confusion matrices of the classification. Representative examples from Animals 1 and 4 are presented in figures 4 and 5, respectively.

The presented scatter plots correspond to the pair of electrodes that resulted in the lowest cross-validation error when used as inputs to the classifier. In figure 4, where the 16-channel cuff was placed proximally, no clear separation between most of the classes on either the 3- or the 16-channel cuff was observed in the feature space. The only exception was the  +30 proprioception class on the 16-channel cuff. This is further confirmed by examining the confusion matrix where the  +30 proprioceptive class was better identified when information was provided from the 16-channel cuff. In all cases, when either the 3- or 16-channel cuff was used, a separation in the magnitude of the proprioceptive angle was seen as a result of an increase in MAV on all electrodes. Similarly, a difference between the heel and outer toe pinch was observed as an increase in MAV on all electrodes when the heel was pinched compared to when the outer toe was pinched. No obvious separation can be identified for the touch stimuli presented in any of the experiments. When the 16-channel cuff was placed distally, a difference in the ratio of the MAVs can also be observed (figure 5(a)). This was only observed in scatter plots where the 16-channel cuff was placed distally. This corresponded to an increase in the correct identification of the proprioceptive classes as shown in the confusion matrix.

### 3.2. Comparison of distal and proximal implantation

In Experiment 1B, we noticed that the benefit of using the 16-channel cuff was greater when the 16-channel cuff was placed distally. Thus, to ensure this difference was due to cuff placement rather than differences in the cuff itself, in Experiment 2 we implanted two 16-channel cuffs, one distally and one proximally.

**Figure 6.**
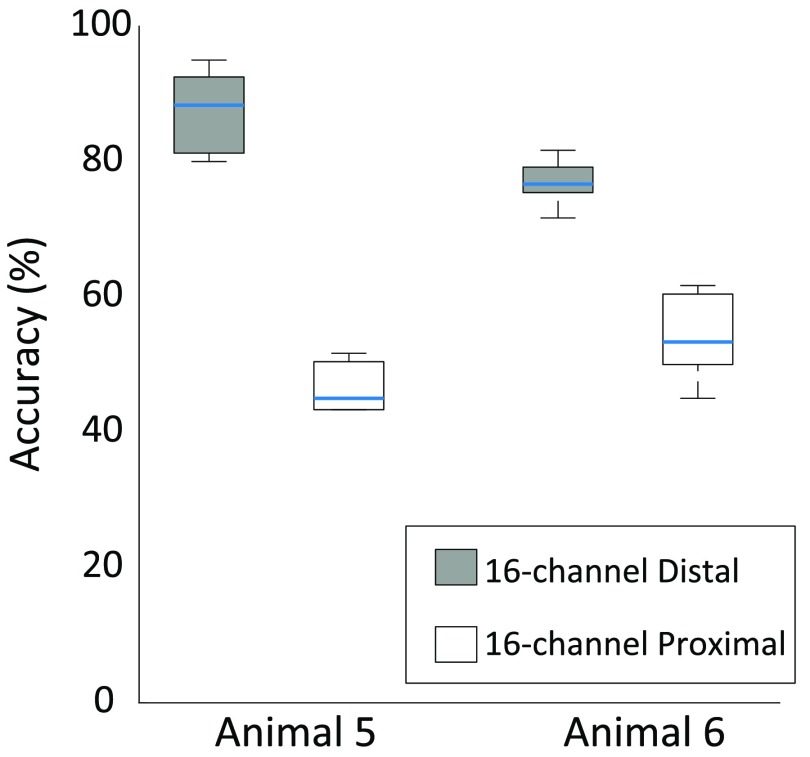
Cross-validated classification accuracy obtained in Experiment 2 for Animals 5 and 6. In both animals, classification of the ENG signals recorded by the distal 16-channel nerve cuff led to higher classification scores. Box plots show median scores across six classes within each animal. Straight lines, medians; solid boxes, interquartile ranges; whiskers, overall ranges of non-outlier data. Chance level: 16% (six stimulation classes).

In both Animals 5 and 6, the median cross-validation accuracy achieved was larger when information from the distal cuff was fed to the classifier rather than when information from the proximal cuff was used (figure 6). The increase in the median cross-validation accuracy with the distal compared to the proximal cuff was 43.3% and 23.3% for Animals 5 and 6, respectively.

**Figure 7.**
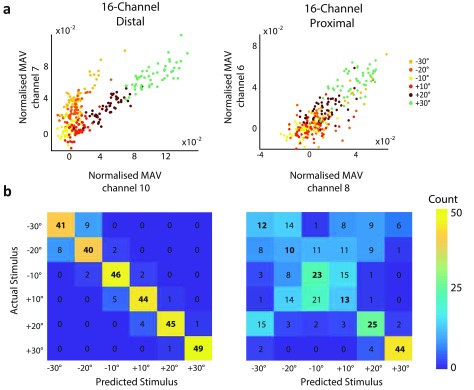
Results obtained in Experiment 2 for Animal 5. (a) Scatter plots presenting the best performing pair of features for the distal (left) and proximal (right) cuffs; (b) Confusion matrices of the cross-validation using the information from the distal (left) and proximal (right) cuffs.

The scatter plots and confusion matrices presented in figure 7 illustrate the results obtained when the 16-channel cuff was placed distally and proximally for Animal 5. The scatter plot of the feature space from the proximal cuff of Animal 5 shows a similar distribution to the proximal cuffs in Experiment 1, whereby an increase in the magnitude of the angle results in an increase in MAV on all electrodes and no obvious distinction can be made between the different angle directions in the feature space. By contrast, examining the scatter plot generated using the distal cuff, it can again be observed that the ratio of the MAVs on the two electrodes is different for positive angles versus negative angles (figure 7(a)). This corresponded to an increase in the correct identification of the proprioceptive classes.

**Figure 8.**
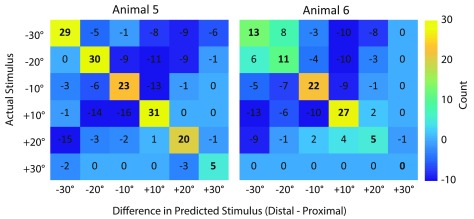
Confusion matrices of the difference between the cross-validated results of the distal cuff minus the proximal cuff for Animals 5 (left) and 6 (right).

Finally, for completeness, we report the difference between the confusion matrices obtained with the information from the distal and the proximal cuffs for both Animals 5 and 6. Figure 8 confirms that in both animals, the distal 16-channel cuff outperformed the proximal 16-channel cuff.

## 4. Discussion

We aimed to answer two questions. Firstly, whether increasing the complexity and consequently the cost, of a nerve cuff would significantly improve the separability of the ENG data. Secondly, whether the position of the nerve cuff affects the separability of the recorded ENG signals. Afferent ENG signals were recorded in response to mechanical stimulation of the hindpaw from nerve cuffs implanted on the sciatic nerve. The recorded data was used as input to a classifier and the resultant performance of the classifier compared under different nerve cuff configurations. The results suggest that it may only be worthwhile to increase the complexity of a nerve cuff if the cuff can be implanted distally, adjacent to the nerve branching.

**Figure 9.**
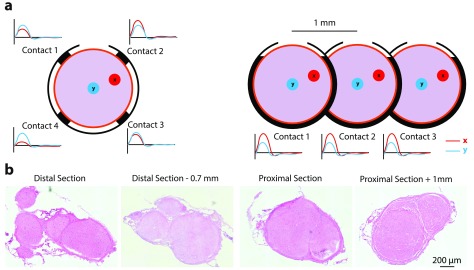
(a) Schematic representation of the sciatic nerve with one ring of the 16-channel cuff (left) and the three ring contacts of the 3-channel cuff (right). The ring of the 16-channel cuff contains four contacts that will record differently according to their relative distance to individual nerve fibres (represented in red and blue by *x* and *y*). The three ring contacts of the 3-channel cuff are placed 1 mm apart from each other in the cuff, therefore, the information collected by each ring contact will be similar. The blue and red lines are a conceptual illustration of data that would have been recorded by the contacts. (b) Histology sections of the rat’s sciatic nerve. On the right two proximal sections (1 mm apart from each other) and on the left two distal sections of the same sciatic nerve. In the distal sections, the division into the different nerve fascicles is more accentuated than in the proximal sections.

In all cases, the classifier performed with an accuracy greater than chance and different classes could be identified as changes in MAV recorded on all electrodes. Nevertheless, the greatest classification accuracy was achieved when the classifier was fed with information from a 16-channel cuff placed distally. In these cases, when the classifier was fed with information from a distal 16-channel cuff, scatter plots of the feature space indicated the classes were not only separated by an increase in MAV on all electrodes but also by a change in the ratio of the MAV between the electrodes (figures 5(a) and 7(a)). A greater understanding of what might be happening can be achieved by examining the underlying anatomy. The rat sciatic nerve is unifascicular at the trochanter, where the femur connects to the hipbone. However, about 5–7 mm distally the nerve splits into two fascicles, namely, the tibial and the peroneal. Then, the tibial portion divides into the sural and the tibial nerves while the peroneal portion gives origin to the peroneal nerve and the cutaneous branch [34]. These sections can be observed in figure 9(b). However, on the proximal sections of the sciatic nerve, only the tibial and peroneal fascicles are distinguishable. This increase in fasciculation distally most likely contributes to the increased classification scores seen when the 16-channel cuff is placed distally. This result is in agreement with the untested hypothesis in [35] that suggested that placing nerve cuffs distally will improve their selectivity. Although, when implanting a nerve cuff it is also important to consider the location of the implant in terms of how stable the cuff will be with repeated movement of joints. Thus, it may still be desired to place cuffs proximal to joints as this may improve surgical accessibility and the long-term stability of the implant [35].

This leads to the question of why then does the performance of the 3-channel cuff not correspond to its location on the nerve. The difference in performance between the two types of cuffs stems from their different geometries. The 16-channel cuff included four rings with four contacts placed circumferentially around the nerve, while the 3-channel cuff included only three ring contacts. On one hand, with the 16-channel cuff, the four contacts of each ring placed at different sides of the nerve could sense the different signals coming from nerve fibres nearby (figure 9(a)). On the other hand, the ring contacts of the 3-channel cuff recorded an average of the neural signals propagated on that site of the nerve. The ring contacts were only 1 mm apart from each other, and histology showed that within 1 mm the spatial layout of the nerve does not change substantially (figure 9(b)). Hence, each of the ring contacts was likely to be recording similar signals. This could be the cause of the poor performance of the 3-channel cuff. This is in agreement with Rozman *et al* [36] where a selective recording was achieved by positioning the electrode contacts over the surface of the nerve so that it registered the signal of a different fascicle more strongly.

These results demonstrate that considerably higher classification scores can be achieved if the underlying nerve structure is considered before implantation. This finding may translate into increasing the selectivity when other neural interfaces, such as flat cuffs, transversal or multi-aisle electrodes [17, 37, 38] are used. For example, corroborating our finding, Freeberg *et al* [37], placed their composite flat interface nerve electrodes (C-FINE) proximal to the tibial and common peroneal branches of the sciatic and showed acceptable selectively. Their approach is conceivable since the working principle of these electrodes is flattening the nerve bundles. In other work, the TIME interface [17] was shown to selectively record information from different subsets of axons thanks to its intrafascicular characteristics and the multiple contacts. However, the positioning of the TIME along the nerve was not investigated. According to our findings, it is predicted that implantation of the TIME interfaces as distally as possible in the sciatic nerve can improve the discrimination of the afferent signals.

We demonstrated that different classes of afferent sensory data can be separated by using the MAV feature of the signal and a linear classifier. However, the time window chosen for feature extraction was 0.5 or 2.5 s only and classification was only examined for signals collected during the steady state. Further work is still needed before such a classification algorithm could be used for real-time application. Importantly, we would need to determine whether a smaller moving window could be used for feature extraction to improve the resolution of the device. While real-time classification is beyond the remit of this paper, offline classification is useful to inform the design and positioning of neural interfaces, in this case, nerve cuff electrodes.

Translating the present study into human studies would require special attention when choosing an adequate neural interface for signal recording. The human sciatic nerve comprises a much higher number of fascicles (25–70) than the rat sciatic nerve (less than 5) [39, 40]. A modelling study by Raspopovic *et al* [41] demonstrated that the optimal neural interface is dependent on the nerve section to be implanted, therefore the optimal design of a neural interface for a human would most likely be significantly different than that for a rat given the differences in fascicle count and distribution. It would be expected that a neural interface to be used in a human would incorporate a high number of electrode contacts placed circumferentially around the nerve so that each electrode contact was located close to a different fascicle. Alternatively, several nerve cuffs could be implanted on different nerve branches, although this could greatly increase the complexity and invasiveness of the surgery.

Recent studies have shown that it is possible to provide sensory feedback to prosthetic limb users by stimulating the peripheral nerves [9, 19]. The results of our study may also help to inform the placement of electrodes for stimulating peripheral neural interfaces, although being able to record selectively does not always translate to being able to stimulate selectively. Tan *et al* [9] were able to provide natural touch sensation on different sites of the subject’s prosthetic hand with implanted cuff electrodes for more than a year. The stimulation selectivity achieved was considered very high since almost all of the channels used for stimulation produced either a unique sensation or sensation in a unique location. The neural interfaces used for their study included two eight-contact FINE cuffs and one four-contact CWRU (Case Western Reserve University) spiral electrode, having a total of 20 stimulation channels in one subject and 16 channels in a second volunteer. In a different study by Wendelken *et al* [19] two USEAs of 100 channels each were implanted on humans for restoring motor control and sensory perception. In the Utah array study, a total of 131 different percepts were reported by the subjects in the form of proprioceptive and cutaneous sensations in the virtual hand. These studies indicate that the selectivity of cuff electrodes may not ever rival that of the intrafascicular arrays. Nevertheless, cuff electrodes still have the best track record for long-term stability and noise resistance.

### 4.1. Conclusions

We assessed the performance of a classifier fed with information from either a 3- or 16-channel nerve cuff, implanted either proximally or distally along the sciatic nerve. This work demonstrates that increasing the complexity of a nerve cuff by increasing its channel count may only be valuable if the nerve cuff can be implanted distally. This takes advantage of the fasciculation of the nerve and makes it possible to identify information travelling along different fascicles more easily. Therefore, this study highlights the importance of considering the anatomy of the nerve before designing and implanting a nerve cuff electrode.

## Open access statement

Upon acceptance, this work will be made Open Access, under the CCBY Creative Commons license. The ENG data supporting this publication and additional metadata will be made freely available via the Newcastle University Research Data Service.
